# Supplementary material for: COZOID: contact zone identifier for visual analysis of protein-protein interactions
Source: BMC Bioinformatics. 2018 Apr 6;19:125. doi: 10.1186/s12859-018-2113-6 (PMC5889581; doi:10.1186/s12859-018-2113-6)
Supplement: Supplementary file 2 — Software build. Executable binary file of the software tool. (ZIP 118,784 kb) [file 12859_2018_2113_MOESM2_ESM.zip › cozoid/platform/docs/testng-6.5.1-javadoc.zip!/org/testng/IReporter.html]

IReporter (TestNG)


---


|  |  |  |
| --- | --- | --- |
| **Overview** \| **Package** \| **Class** \| **Tree** \| **Deprecated** \| **Index** \| **Help** | |  |
| **PREV CLASS**   **NEXT CLASS** | **FRAMES**    **NO FRAMES**     **All Classes** |  |
| SUMMARY: NESTED \| FIELD \| CONSTR \| METHOD | DETAIL: FIELD \| CONSTR \| METHOD |  |


---


## org.testng Interface IReporter

**All Superinterfaces:**: ITestNGListener

---

``` public interface IReporter extends ITestNGListener ```

This interface can be implemented by clients to generate a report. Its method
generateReport() will be invoked after all the suite have run and the parameters
give all the test results that happened during that run.

**Author:**
:   cbeust
    Feb 17, 2006

---

| **Method Summary** | |
| --- | --- |
| `void` | `generateReport(java.util.List<XmlSuite> xmlSuites, java.util.List<ISuite> suites, java.lang.String outputDirectory)`             Generate a report for the given suites into the specified output directory. |

**Method Detail**

### generateReport

```
void generateReport(java.util.List<XmlSuite> xmlSuites,
                    java.util.List<ISuite> suites,
                    java.lang.String outputDirectory)
```

:   Generate a report for the given suites into the specified output directory.


---


|  |  |  |
| --- | --- | --- |
| **Overview** \| **Package** \| **Class** \| **Tree** \| **Deprecated** \| **Index** \| **Help** | |  |
| **PREV CLASS**   **NEXT CLASS** | **FRAMES**    **NO FRAMES**     **All Classes** |  |
| SUMMARY: NESTED \| FIELD \| CONSTR \| METHOD | DETAIL: FIELD \| CONSTR \| METHOD |  |


---
